# An agenda for the medical humanities and ageing

**DOI:** 10.1177/09526951251347503

**Published:** 2025-07-09

**Authors:** Martina Zimmermann

**Affiliations:** 4616 King's College London, UK

**Keywords:** age studies, bio-psychosocial approaches, critical gerontology, medical humanities, policy

## Abstract

Pessimism about ageing is grounded in a decline narrative that upholds youth as embodying the productive, independent self. Challenging this narrative is central to age studies and essential to critical gerontology. The medical humanities, by comparison, have not sufficiently engaged with ageing to date. Rapid demographic shifts across the 20th century, partly brought about by medico-technological progress, have led to a growing number of older people with multiple, chronic, and degenerative health conditions. This, I will argue, is where the medical humanities have a duty to intervene, because their purview and potential is different from that of age studies. The social practices to which illness gives rise are the central object of the medical humanities, and bio-psycho-social factors have had a place in this field since its inception. Embedded in a historical account of the relation of the medical humanities to ageing, and an account of the history of age studies, this article asserts that the medical humanities need to invest their discipline-crossing capacity and knowledge base in ageing. They need to integrate the biological reality of ageing into their descriptive practices and accept biomedicine as a constructive agent in their future work on ageing. This will enhance the field's enormous potential, at the level of policy and practice, to improve ageing for all.

## Introduction

Culturally prescribed ageing is successful ageing, and any deviation from this normative script furthers pessimism about getting older. This pessimism is grounded in a decline narrative that upholds youth as embodying the productive, independent, vital self (Freeman, 2010: 176; Gullette, 1997: 4–5). Challenging this narrative is central to age studies and essential to critical gerontology with its qualitative investment in the social and behavioural realities of ageing. The medical humanities, defined as ‘any attempt to answer a question in which medicine has a stake using ideas and frames of references drawn from the humanities’ (Vickers, quoted in Arts and Humanities Research Institute, 2020: 20), by comparison, have not sufficiently engaged with ageing to date. Rapid demographic shifts across the 20th century, partly brought about by medico-technological progress, have led to a growing number of older people with multiple, chronic, and degenerative health conditions. This, I will argue, is where the medical humanities have a duty to intervene, because their purview and potential is different from that of age studies. The social practices to which illness gives rise are the central object of the medical humanities, and bio-psycho-social factors have had a place in this field since its inception. A stronger embeddedness of biology within the medical humanities would enable a more effective focus on social and psychological factors that contribute to ill health across the lifespan, factors that can be influenced by health and care policy and practice. Set within a historical account of the relation of the medical humanities to ageing, and an account of the history of age studies, this article asserts that the medical humanities need to invest their discipline-crossing capacity and knowledge base in ageing. They need to integrate the biological reality of ageing into their descriptive practices and accept biomedicine as a constructive agent in their future work on ageing. This article therefore argues that the medical humanities’ perhaps somewhat exaggerated concern about the biomedical (Whitehead and Woods, 2016: 15) lessens the field's enormous potential, at the level of policy and practice, to improve ageing for all.

The biomedical itself is not the reason for non-care or un-care. Biomedicine evolved during the second half of the 20th century, when medicine and biology moved closer and closer together, both in institutional terms and at the level of intellectual exchange (Clarke *et al.*, 2003; Keating and Cambrosio, 2003). In parallel, a rapidly growing specialization contributed to medical problems of old age being allocated to disease-specific concepts (Moreira and Palladino, 2009: 357). This focus on specific pathologies has led to ideas that the search for a cure determines how society thinks about the role of medicine in older age (O’Mahony, 2017). Considering older age as a period in life marked by disease and decline has significant implications for experiences and anticipations of ageing at the individual and population level. Taking a bodily state as illness rather than a new normal that comes with increasing frailty in older age potentially is a powerful move because illness seems to hold out the hope, even expectation, of betterment and cure (Clare, 2017: 86). But where a cure is not forthcoming, as in chronic degenerative conditions, ageing is seen as a failure by individuals with significant implications for allocation of resources at population levels.

Older age *does* come with an increased susceptibility to a range of pathologies – cancer, cardiovascular disorders, and degenerative joint and neurological diseases. It is not surprising, therefore, that biomedical research approaches ageing with therapeutic intent (Post, 2000), some even taking it for a disease in need of a cure (Farrelly, 2008). But a critical focus on the biomedical as aligning old age with disease distracts from a whole range of other scientific endeavours, especially biological senescence research. This field studies the mechanisms underlying biological, physiological, and functional ageing. It treats ageing as a lifetime continuum involving ‘growth, development, and maturation … just as much as atrophy and degeneration’ (Shock, 1951: 1; see also Baker and Achenbaum, 1992). Alongside material changes of the body, varying levels of energy, and the possibility of ill health, biological ageing both accommodates and limits the continuing potential of older people.^1^ A stronger awareness of biological ageing as reality has the potential to shift attention to modifiable factors, especially social and psychological elements; and this would align with the medical humanities’ growing investment in social justice, not least since addressing social and psychological elements can delay biological ageing.

I seek in this article to develop an agenda for the medical humanities to critically reassess its relationship to the health sciences, including biology and biomedicine, and to radically extend its long-standing investment in bio-psycho-social approaches to concerns about health and disease in older age. The stories about ageing we listen to interlock biological, psychological, and social aspects of ageing. As a result, my proposal comes with an encouragement to reappraise the value of narrative for ageing – as a tool for study, as a means of care, as a driver of health. The fragmentation of medical knowledge since the 1930s has contributed to reifying experiences in the clinical encounter, as the patient's body became more and more compartmentalized. Historically, narrative approaches have been of enormous value in drawing attention to the experience of ageing, including aspects of illness, vulnerability, and dependence and their impact on shifting identity. Critical medical humanities endeavours have importantly pointed to the normative implications of narrative approaches to health and disease but work on ageing has mainly focused on aspects of care in a global economy (Woods, 2011; Whitehead and Woods, 2016: 17–18); narratives about ageing, particularly those reflecting the ‘plurality of scenarios for later life’ (Misztal, 2020: 1; see also Hepworth, 2000; Zeilig, 2011), are near absent from recent medical humanities research.^2^ I advocate for a reassessment of the value of narrative for accessing the diverse experiences of ageing and I recommend that the medical humanities engage more fully with biological approaches to ageing, as these further attention to the impact of cumulative inequalities on health in older age.

I am suggesting that, once it is embedded within the medical humanities, biological senescence research has significant potential for improving lifelong ageing for all, not least since biomolecular substrates tell stories that synergize with narrative evidence and concerns. To develop this argument, I will explore two areas. First, I will attend to the use of narrative in age studies and gerontology. I will summarize past and current scholarship on ageing and focus on how narrative has mattered to gerontology for understanding the experience of ageing and championing care in the context of its social and behavioural aspects. Concurrently, I will question the role of narrative and its temporal aspects in contributing to accounts of degeneration and decline, while exploring its potential in supporting identity-affirming decisions in elder care. Second, I will make the case for the medical humanities to engage in biology. I will explore how a greater negotiation with biological senescence research could reinvigorate the medical humanities’ long-standing concern with health and care; alongside, I will illustrate the flaws of an exaggerated critique of the biomedical. In making the case for a ‘biological turn’, I will suggest that the field's concern with the bio-psycho-social pathways of ill health is not radical enough and sufficiently focused on ageing; that a closer research connection between the biological sciences and the health humanities is not an opportunity, but a necessity, if the field is to be genuinely invested in healthier ageing for all.^3^ To this end, I will draw attention to scientific evidence that shows how culture determines health in ageing via biological, psychological, and behavioural pathways.

Overall, my arguments for ageing to become central to the medical humanities are concerned with the older person and their care in a society overly focused on curative solutions. Observing a partly flawed societal, political, and economic investment in biomedicine, I interrogate the role of culture and society in driving research priorities that favour cure over care. Concurrently, I explore health in ageing as a product of the social, cultural, and technological, identifying biology as tracing the impact of these spheres on health outcomes. Engaging with bioscientific research that spells out the reality of the human condition, while offering clear targets for intervention at a cultural, societal, and economic level, holds the potential for the medical humanities to influence policy and practice for an ageing population more effectively.

## Narrative in age studies and critical gerontology: Lessons for the medical humanities

Age studies began to form during the 1970s. Since their inception, the field has considered biological ageing as mediated by cultural construction (Gullette, 1997: 154). Grounded in political and aesthetic engagement, it probes age as an aspect of identity, modelled on gender studies (Pickard, 2016: 1–25). Initially, the field was dominated by historical, religious, philosophical, and humanistically invested social science scholarship that essentially addressed ‘basic moral and spiritual issues’ around ageing (Cole, 1992a: xi). Concurrently, age studies strongly benefitted from the relationship between the humanities and gerontology, since narrative has been essential to both age studies and gerontology (Katz, 2016: 220). Gerontology defines itself as a science, centred around the biology and biomedicine, psychology, and sociology of ageing (Bengtson, Putney, and Johnson, 2005: 16). But from this initial emphasis gerontology has come to borrow from a range of disciplines across the human sciences and the humanities, including anthropology, economics, epidemiology, history, political sciences, psychology, social work, and the arts and humanities (Achenbaum, 1995; Kolb, 2014: 15). Beginning in the 1960s, the life review initiated the use of narrative approaches in gerontology (Butler, 1964; Weiland, 1993: 84). Narrative became ‘an essential form of seeking and representing knowledge’ in relation to growing older (Cole and Ray, 2010: 10; Cruikshank, 2013: 197–200), and, by the 1980s, narrative models complemented quantitative work, illuminating the social and behavioural aspects of ageing in the field of critical gerontology. How did this happen?

During the 1980s, quantitative studies were the gold standard in gerontology. They harnessed data that emerged from the large-scale longitudinal studies launched during the late 1950s and early 1960s (Park, 2016: 211–45). Such quantitative work assessed care needs or intellectual capacity with numerical tools such as the Activities of Daily Living Scale or the Blessed Dementia Scale, but in doing so cultivated a decline narrative (Burack-Weiss, 2015: 9–10). Concurrently, a key endeavour of these longitudinal studies had been to determine what was normal and what was pathological in older age to identify ways to prolong health in ageing (Achenbaum, 1995: 76–81; Park, 2016: 227; Shock *et al.*, 1984: 63). For the purpose of describing ‘changes which occur in the absence of diagnosable disease states’, these studies sought out individuals who matched the researchers’ ideal of health in old age, that is, individuals living ‘under conditions in which economic status and educational level would not seriously limit medical care, nutrition and other factors affecting health over the life span’ (Shock *et al.*, 1984: 52). As a consequence, these studies largely relied on chronological age for sampling and conflated the body of the white middle-class adult male with the idea of the ‘standard human’ (Epstein, 2007: 78; Moreira and Palladino, 2011: 318; Park, 2016: 231, 240). Hand in hand with these practices went an assumption that people became more similar with age, meaning that attributes such as race, ethnicity, sex, and class legitimately could be – and were – neglected in their effect on individuals as they age (Kolb, 2014: 13).

The concept of *successful ageing* originates from this period. Sociologists Rowe and Kahn (1987) had introduced it to overcome the acceptance of disease states in older age. But reflecting the spirit of longitudinal research to define normal ageing, successful ageing emphasized ideals such as individual achievement, productivity, and autonomy, without acknowledging that ageing comes with real changes of the body – changes that don’t fit into a normal/pathological or active/passive dichotomy (Lassen and Moreira, 2014: 44; Liang and Luo, 2012; Sandberg, 2013; see also Ciafone, 2017: 156). This created pressures for older people. The realization that much of the biological ageing process is outside an individual's control can leave them feeling powerless; and not achieving successful ageing comes with perceptions of failed ageing (Calasanti, 2016: 1093; Katz and Calasanti, 2014). This is important because gerontologists themselves bring their own preconceptions (Featherstone and Hepworth, 1991: 381).

Critical gerontology formed in response to these developments, as ‘a style of inquiry rooted in practice’ (Cole, 1992a: xxiii; Achenbaum, 1992). Pointing to the shared agenda with the medical humanities, the first conference on critical gerontology was held at the Institute for the Medical Humanities, Galveston, Texas, in January 1991. The relevant scholarship arose from the social sciences, clinical medicine, nursing and social work and employed qualitative research methods rooted in the humanities (Cole and Ray, 2010: 5). Humanities disciplines were seen as promoting a ‘more intellectually rigorous gerontology’ – by suggesting new hypotheses for empirical research, by revealing values, assumptions and relationships in findings arising from empirical work, and by pointing to the role of cultural settings in shaping values and intentions (Cole, 1993: vii–viii). In this context, narrative approaches became essential during the 1980s and 1990s, because they were thought to provide ‘the nuance, complexity, contradiction, and incongruities’ of older age not captured in quantitative research (Cruikshank, 2013: 197; see also Burack-Weiss, 2015: 14).

Critics of narrative gerontology doubted the resemblance of narrative to reality (Kolb, 2014: 31). But the life story has created tremendous opportunities for study across the humanities and social sciences. As behavioural scientist Bertram J. Cohler points out,

Examination of the life-story construct, using the concepts of interpretation and criticism developed by the humanities, together with the methods and findings from social science studies of lives over time, has brought about a renewed appreciation of the significance of meaning and coherence, as well as the role of memory and present experience, in laying the foundations for an individual's life-story construct, or personal narrative. (1993: 108)

The interpretive work of the humanities has supported the appreciation of the person as a ‘sociohistorical and sociocultural product’ (Berman, 1994: 18, 33), and the parallel rise of narrative medicine further underwrote a focus on experience in the clinical encounter (Brody, 2003; Hunter, 1991; Kleinman, 1988, 2019; on the centrality to the medical humanities of the individual's point of view, cf. Rashed in this issue). But a focus on ageing and degenerative chronicity was slow in coming, as prominent interventions in narrative medicine, among others, centred on cancer, HIV/AIDS, and physical disability (Couser, 1997; Hawkins, 1999).

Literary scholarship contributed systematically to these conversations from the mid 1970s, expressed in a growing number of qualitative studies on literature published in the *Gerontologist* (Wyatt-Brown, 1992: 331–2; for excellent histories, consider Cole, 1992b; Small, 2007; Thane, 2000). Taking stock of life writing that has been growing since the 1970s, literary studies have sought to explore ageing as an experience, while challenging the culture-led narrative of decline (Oró-Piqueras and Falcus, 2018: 3; for an influential contribution on ageing in film, see Chivers, 2011). In addition, literary scholarship helped to amplify the voices and perspectives of marginalized groups, including people with dementia (Bitenc, 2020; Burke, 2007; DeFalco, 2010; Falcus and Sako, 2019: 26–79; Goldman, 2017; Zimmermann, 2017; 2020: 123–9). As part of this, an important aspect of this scholarship has been to explore the opportunities and challenges related to the narrativization of ageing (Barry with Skagen, 2020: 4).

Concerns about narrativity had crystalized with the turn towards a narrative paradigm in the social sciences and medical practice.^4^ An emphasis on narrative became problematic from the moment when specialists like clinicians professed a stake in narrative, that is, when a high level of narrative articulacy was considered essential for a productive clinical encounter (Charon, 2006; Goodson and Gill, 2011). Beyond the ability to narrate, normative claims have encompassed aspects of narrative form and performance. This includes the assumption that narrative construction of meaning is central to how people experience their lives. In addition, specific versions of an illness experience were seen as fashioning the narrator as a survivor of their condition, restored to their former life (Frank, 1995; Hawkins, 1999). Such resilience narratives were deemed indicative of an individual's ability to ‘rise above their suffering, battle their disease, and believe that everything will be fine in the end’ (Conway, 2007: 18). Yet such a position is not available to a person with a chronic degenerative condition like arthritis or dementia, nor does it support older people more generally as they navigate changes in their bodies.

Critical gerontology and age studies have long since critiqued such resilience narratives, as they reflect thinking patterns that chime with notions of successful ageing and, linked to this, expectations kindled by the biomedicalization of ageing. The biomedicalization of ageing is a process enabled by medico-technological progress, which brought ageing into the scope of medicine, with repercussions for scientific research, clinical approaches, policy focus, and public perceptions of ageing (Estes and Binney, 1989). Already from the 1930s onwards, health care had become increasingly organized around responses to specific diseases (Carboni, 1982: 120–7; Moreira and Palladino, 2011: 320). By the 1950s, conditions and problems involving care for older people had moved to the domains of medical specialties, reflected in how specific sections of the National Institutes of Health became focused on diseases (Hirshbein, 2000: 346). This development opened biological processes of ageing to curative intervention, in the form of treatment, enhancement, or enrichment that pushed ageing away ‘from a natural life event to a medical problem’ (Conrad, 2007: 23; on the problem of ageing as ‘subsumed to illness as event’, see also Malabou, 2012: 39).^5^

What all of this means is that cultural discourse is invested in a dichotomy of young versus old, where medicine is both imagined and expected to extend the period of youth. Especially since the second half of the last century, medico-technological progress has resulted in a steady prolongation of life. But this was not necessarily accompanied by an increased number of years spent in health. More and more older people struggle with chronic degenerative conditions. Concurrently, medico-technological progress has contributed to the devaluation of ageing as an experience. It encourages belief in the success of complicated interventions at later and later ages (Kaufman, Shim, and Russ, 2004). These developments obfuscate the fact that, throughout life, health is a dynamic state that is continuously ‘subject to change and revision’, depending on context and environment (Canguilhem, 1991[1978]: 209; 294, n. 32; Margree, 2002: 301–2; Tiles, 1993: 737; see also Bunting, 2021[2020]: 228; Pickard, 2018). Medical technologies suppress appreciation that, with increasing age, health becomes more difficult to maintain (Dreuil and Boury, 2010; Giroux, 2012: 49–50). This is also reflected in how end-of-life decision making becomes more and more challenging: a ‘medical lens’ is used to understand the distress of an old and frail person (Kaufman, 1998: 715, 721), meaning that quality of life may become neglected in an effort to extend or maintain life (Gawande, 2015[2014]; O’Mahony, 2017). As a result, degenerative chronicity in old age has blurred the lines between ageing and illness (Lafontaine, 2009). These developments warrant a critique of the biomedical.

In summary, narrative approaches were instrumental in the social sciences for the clarification of aspects of ageing that quantitative research could not capture; they also helped articulate ageing as a ‘complex process where continuity and change take place in the context of knowledge of finality and the increasing bodily vulnerability’ (Misztal, 2020: 14). But they harboured the danger of fostering, rather than challenging, a narrative of decline in relation to ageing, underwriting problematic notions of successful ageing that envision dualities of healthy versus sick and normal versus pathological. Next to critical gerontology and age studies, critical disability studies, with their scepticism of cure, have articulated concerns about ableism as the ‘obstacle to a good life’ (Kafer, 2013: 2), stressing that disability is the one identity category that everyone will inhabit if they live long enough (Davis, 2002: 1–8; Garland Thomson, 2017[1997]: 13–14; McRuer, 2006: 30; see also Murray, 2023: 61). The medical humanities, in turn, have highlighted the problematic implications of resilience narratives (e.g. Conway, 2007; Diedrich, 2007; Jurecic, 2012). Alongside, the critical medical humanities have been important for raising concerns about the limits of narrativity (see esp. Woods, 2011; cf. Vickers in this issue on narrative as an enduring concept), emphasizing the value of chaos and episodic narratives for example in the context of chronic conditions including dementia (e.g., Bitenc, 2020; McKechnie, 2014; Ward and Sandberg, 2023; Wasson, 2018; Zimmermann, 2024). But the critical medical humanities’ turn away from narrative approaches have perhaps inadvertently contributed to an absence of narratives about ageing in recent medical humanities research and a sidelining of experiential aspects of ageing in the field. A focus on race, class, gender, and sex has questioned the ‘uneven flows of knowledge, power, bodies, expertise and organs’ in a global context of health and care (Whitehead and Woods, 2016: 17). Such work has highlighted the asymmetry between the carer and the cared-for in a global economy (Ciafone, 2017; Woodward, 2012; cf. also Schwering in this issue). But in their scope, these efforts have neglected an appreciation of how these categories of difference impact on health in ageing.

In other words, the medical humanities have increasingly focused on social justice, but ageing has featured very little among discipline-crossing and intersectional research topics. In addition, the medical humanities have the potential to contribute with a more nuanced appreciation of the value of narrative, because narrative is essential to a critique of the implications for older age of biotechnological progress. Sociologists like Berman point out how ‘meaning-making is inextricably linked to cultural *and* biological givens’ (1994: 64; original emphasis), and here lies an open area of investigation for the medical humanities. The approach of biological senescence research to ageing as a lifelong process overlaps with values of narrative concepts. As bioethicist Nancy S. Jecker explicates, ‘Understanding life as a narrative best explains how people can undergo dramatic changes yet persist over time as protagonists in one and the same story.’ Jecker uses narrative to understand life as organized, as a means that is able to ‘unify life's moments’. This is particularly important for making ‘ethically sound decisions’ on behalf of people who can no longer speak for themselves, argues Jecker, as their past, present, and future selves belong to the same story (2020: 99, 123).^6^ Moreover, as I explore further in the next section, biological ageing captures the impact of lifelong inequalities connected to the categories of difference that the medical humanities focus on.

## An expansion of the medical humanities: A ‘biological turn’ enables focus on social and psychological factors that age us

Turning to the biological realities of ageing can help achieve productive shifts in how the medical humanities approach ageing. Biogerontological endeavours invested in understanding the biology of ageing rather than targeting specific diseases of older age understand themselves as an alternative to the biomedical. Since the 1980s, this area of senescence research has pursued the idea that cancerous and degenerative age-associated conditions share basic cellular and molecular processes identified as responsible for old age. An understanding of these mechanisms is central to the biogerontological endeavour (Olshansky, 2019: 117). In other words, long-term chronic conditions common in older age are ill addressed by research on discrete aetiologies. Arguments about ageing as natural have been put forward by evolutionary biologists and favoured by gerontologists (Giroux, 2012: 41–3; Moreira, 2017: 183–4). Reformulating gerontology with evolutionary biology in mind would accept that ageing is not a ‘universal biochemical mechanism’, which could be uncovered if one searched hard enough (Rose, 1994[1991]: ix; Rose and Graves, 1989). It would recognize evolution as the cause of ageing in multicellular organisms. In addition, it would enable considerations of the experience of ageing in affluent societies as shaped by the misalignment between the fast medico-technological, societal and cultural evolution of the last 200 years and piecemeal human evolution (Singer, 2019: 18–24). It would strengthen productive work on how biological, psychological, and social factors determine health in ageing.

In line with these observations, it is perhaps not surprising that some of the more enabling experiential accounts of ageing come with an acceptance of the human condition and its biological evolutionary reality. In *Growing Old: Notes on Ageing With Something Like Grace*, for example, American anthropologist Elizabeth Marshall Thomas reflects on the ‘unlimited opportunities’ of natural selection (2020: 11). Observing the evolutionary path of different ‘life-forms [that] are still on earth after three billion years’, Thomas collapses the immortality of the species with the mortality of the individual. She finds that the path of natural selection has made man's ‘journey … more exciting’, the ‘end of our journey appear[ing] as we age’ (ibid.: 12). British writer Penelope Lively points to the principles of natural selection and appreciates palaeontology for the fact that ‘it puts you in your place – a mere flicker of life in the scheme of things’ (2014[2013]: 182, 208). For Lively, an expanded ‘concept of time’ is essential to appreciate the human condition. ‘There is a further dimension to memory’, she writes; ‘It is not just a private asset, but something vast, collective, resonant’ (ibid.: 234). Grounded in the concept of evolution, her vision of collective memory, as Zymunt Bauman would say, serves the living as an ‘anchorage of their desire of immortality’ (1992: 52). The psychologist Florida Scott-Maxwell, in turn, articulates experiences of pain and ‘shaky health’ in *The Measure of My Days* (1979[1968]: 115). Her vision of individuality in the face of frailty and physical losses is grounded in the concept of evolution that develops the argument about immortality formulated by Bauman: expanding life by assigning life-transcending value to specific activities or achievements (1992: 5, 6). For Scott-Maxwell, being an individual is a way ‘to advance humanity’; ‘by living our lives’, she finds, ‘we create something fit to add to the store from which we came’ (Berman, 1994: 153–4; Scott-Maxwell, 1979[1968]: 40).^7^

Biological senescence research describes physiological impact and functional outcomes as following on from biological changes from our 20s (Ferrucci *et al.*, 2018). Such research embedded in evolutionary theories has progressed since the late 1800s, but cultural uptake of such research remains selective. Sociologist Tiago Moreira has scrutinized the position of gerontological research in the critique of the biomedical, finding that biogerontology has become aligned with the prolongevity movement and the anti-ageing industry (2017: 183–9). There are several different conceivable outcomes of senescence research (Juengst *et al.*, 2003), and these complicate the acceptance of biogerontological undertakings. Prolonged senescence as such encapsulates the fears currently projected onto ageing: an ever-increasing lifespan that merely extends the years fraught with age-related ailments. As outlined above, this scenario aligns with the developments witnessed in affluent societies over the last 100 years. In this context, biogerontology is pitched as having the potential to arrest ageing (Anton, 2013). But dreams of arrested ageing fuel further pessimism about ageing because they claim the status of curative solutions. This dynamic diminishes opportunities for accepting the hard biological reality that ageing comes with a ‘progressive loss of physical integrity, leading to impaired function and increased vulnerability to death’ (López-Otín *et al.*, 2013: 1194).

Compressed morbidity towards the end of life, by comparison, would increase the health span. During a limited lifespan, people would live longer without the debilitating chronic conditions that have become the hallmarks of old age. This scenario is envisioned by biogerontologists who target the ageing process as encapsulating a mechanism common to degenerative conditions associated with older age. Take for example the following consideration of research in relation to longevity genes. In collaboration with English scholar Vita Fortunati, immunologist of ageing Paolo Franceschi expounds that

the aim of biogerontologists is to identify such genes [of longevity] and understand their biological role as tools to avoid, or significantly postpone, major pathologies and disabilities associated with old age. Scientists are neither re-proposing the myth of eternal youth nor searching for the elixir of long life; rather, and more modestly, they are combating disease and premature death. (Fortunati and Franceschi, 2011: 191)

This explication underlines biogerontological goals for health and well-being in older age, but it also highlights the continued importance of targeting age-specific diseases, which aligns with the principles of biomedical approaches to older age characteristic of Western cultures.^8^

The above illustrations suggest that targeting the biomedical per se seems misguided. Reflections from bioethics and disability studies confirm this. For sociologist Tom Shakespeare, the medical model has become a holdall ‘for all that is wrong with traditional attitudes to disability’, including medicalization, objectification, and paternalistic research methods; the same is true for how a social science critique of medicine refers to concepts like medicalization and the biomedical model of disease: they reify medicine as a concept (Shakespeare, quoted in Dokumacı, 2019: 166). This, I argue, leads to an insufficient appreciation of specific, differentiated practices and areas of interest in medico-scientific research. The biomedical has a place in the relationship between scientific knowledge, social action and subjective experience, and articulations across these domains, which ‘co-produce’ each other (Pickersgill, 2012: 328). In a marketplace of ideas and hypotheses, society selectively absorbs scientific models and convictions. In addition, professional cultures and healthcare systems shape the implementation of research priorities, treatment programmes, and diagnostic categories (Moreira *et al.*, 2008; Wilson, 2014: 143, n. 18). A critique of the biomedical should take account of these interrelationships and influences.

But in proposing a ‘biological turn’, I am not advocating biological determinism. The likelihood of developing a disease or disability is only partly related to the genes inherited; it is much more affected by social, cultural, and economic factors (e.g. Swynghedauw, 2019: 25–36; Kenny, 2022: 42–5). Mindful of these interconnections, the medical humanities would be strengthened by radically expanding their long-standing investment in the social and behavioural aspects of health and disease. Such work has the potential to contribute to compressed morbidity for all, by challenging lifetime inequalities and their cumulative effect on ill health in older age. In what follows, I scope out some areas for further engagement.

Starting in affluent societies of the 1980s, the progressive privatization of healthcare turned medico-technological progress into a commodity. As a consequence, experiences of ageing became increasingly tied to financial means (Higgs and Gilleard, 2015: 8; Phillipson, 2013: 99), leading to growing disparity in health outcomes. Identities, including the nuclear family, gender, and class, were recognized as strongly influencing how one ages (Katz, 1995: 70; see also Giroux, 2012: 36). This came into sharp relief during the 1980s, as the first generation of post-war immigrants to the UK entered their 50s and 60s. Yet, reviewing the literature at the turn of this century (and again 15 years later), epidemiologist Nancy Krieger observes that there is only scant research that investigates how structural discrimination impacts health across the life course (2014: 643). Biogerontological life course approaches investigate biological, psychological and social risk factor trajectories with an impact on health in ageing (Heikkinen, 2011), and postgenomic research contributes to this body of work, addressing how experiences and environments impact gene expression.

The evidence and meaning of postgenomic sciences, including epigenetics, continue to be highly contested, offering areas for medical humanities investigation. Many consider epigenetics a ‘mechanistic framework for the study of environmental factors in development and evolution’, while others find the value assigned to the field reflects the ‘continued search for confirmation for our social ideologies in the facts of biology’ (Stevens and Richardson, 2015: 4). Needless to say, the biotechnological governance of health pursued by futurologists and venture capitalists invested in multi-billion postgenomic projects perpetuates social injustice, while removing individual control. But since Barker (2004) began developing the ‘fetal origin of adult disease hypothesis’ from the 1980s, epigenetic evidence has increasingly pointed to fetal and perinatal environment as influencing the development of diabetes and heart conditions in later life. In the same way, epigenetic data show that traumatic experiences early in life can lead to physiological and biochemical changes at the level of the genome, with lifelong consequences (Shalev *et al.*, 2013).^9^ There is no question that postgenomic research disciplines and epidemiology have brought evidence from the life sciences, social sciences and the humanities closer together, creating new fora that enable contextualized research (Niewöhner, 2011), which should be harnessed more fully by the medical humanities. Culture, by way of bio-psycho-social connections, has been shown to influence health in older age. I will attend to two specific topics: self-directed pessimism about ageing and its consequences for health in later life, and the cumulative effect of lifetime inequalities on health and well-being in later life. Both have cellular and molecular substrates, speaking to the tight connection between the cultural, social, and biological, thus suggesting how biological research could present a much stronger ally for the medical humanities than hitherto has been acknowledged.

Self-directed ageism has been shown to impact health and well-being outcomes in older age. Epidemiologist Becca Levy has done important work in the field. For example, older adults who hold more negative views about their own ageing are less likely to engage in preventative health behaviours, including exercise and diet; and these behaviours potentially result in worse mobility and physical functioning and an increased risk of cardiovascular events (Levy and Myers, 2004; Levy *et al.*, 2009). Concurrently, individuals with positive self-perceptions of ageing live longer on average than individuals of stigmatized groups (Westerhof *et al.*, 2014). Reviewing experimental and longitudinal studies, Levy has come up with a theory of stereotype embodiment suggesting that stereotypes are internalized across the lifespan, contributing to an understanding, at a biological level, of how ageing is in part a social construct (Levy, 2009). By working together with behavioural scientists and experts in neuroscience to pin down these findings biochemically, Levy shows that negative age stereotypes earlier in life may well predict cognitive performance as well as brain changes associated with Alzheimer's disease (Levy *et al.*, 2011; Levy *et al.*, 2016).

Evidence regarding stereotype embodiment is also relevant for experiences in a global economy. In countries of the Global North, where older people hold low social status, considering oneself as old comes with subjective ill health (Marques *et al.*, 2015), suggesting that cultural indicators have important implications for health and well-being in older age (Levy, 2009). Ageism is much less profound in collectivistic, less affluent cultures, which place more emphasis on interdependence. In such cultures, age bias is much less common, with consequences for subjective health and well-being in older age (Ackerman and Chopik, 2021). Due to migration and colonialism, collectivistic perspectives are alive in marginalized communities of the Global North (Zimmermann *et al.*, 2023), though I don’t mean to pursue an idea of cultural difference as broad and universalizing. Exactly these marginalized communities, aligned with categories of difference including race and class, continue to incur the greatest inequalities in health and well-being in older age, a cumulative inequality that leads to premature mortality (Ferraro, Shippee, and Schafer, 2009).

Thinking in terms of cultural differences is crucial for appreciating ageing-related health inequalities in multi-cultural contexts like the UK, and cumulative inequalities and their impact on health in ageing constitute further potential avenues for exploration by the medical humanities. Reviewing patterns of discrimination, social epidemiologist Nancy Krieger has identified five pathways by which discrimination – racism, sexism, and other forms of social inequality – affect health across the lifespan: economic and social deprivation, hazardous conditions, socially inflicted trauma, targeted marketing of psychoactive substances and other commodities, and inadequate health care (1999: 332). From the mid 1980s, gender, ethnicity, and age have received increasing attention in medicine (Epstein, 2007: 1; see also Kleinman, 2019), but in the second decade of the new century, there remain continued gaps in research that would enable a more ‘nuanced understanding of the likely biological pathways of embodying discrimination, from conception to death’ that must sit alongside a ‘finely tuned historical, social, and political sensibility’ (Krieger, 2014: 697).

Social determinants of health, including money, knowledge, and beneficial social connections, have been proposed as protecting health since the 1990s (Link and Phelan, 1995). In the field of life course gerontology, cumulative inequality theory focuses on system properties that generate inequality (Kolb, 2014: 106). This is in line with approaches, for example, from gender studies and disability studies, which support the recognition of injustice in healthcare systems (Launer and Wohlmann, 2023: 98). Contributing with perspectives from the sociocultural, the medical humanities are excellently placed to encourage and to engage with evidence in the health sciences, including health neuroscience. Well-being goes hand in hand with socio-economic status, and this is reflected in biological correlates that range from the neuroendocrine and immune systems to cardiovascular health and sleep quality (Ryff, Singer, and Love, 2004). Research on cumulative advantage processes suggests, in turn, that health disparities cannot and should not be reduced to health-related mechanisms (including health behaviour, work conditions and medical care), but consider much broader socio-economic contexts from very early in life (Willson, Shuey, and Elder, 2007: 1913). Also in this context, postgenomic data, based on metabolic profiles from multi-cohort analysis, are unequivocal about the impact of social and economic factors on human physiology (e.g. Robinson *et al.*, 2021). The medical humanities should and could go further, genuinely pushing towards a bio-psycho-social description of lifetime inequalities as determining health in older age (for a recent appraisal of the bio-psycho-social model of health and disease, see Bolton and Gillett, 2019; see also Clarke, Ghiara, and Russo, 2019). Knowing about biological realities as one ages can direct attention to social and psychological factors, both of which can be modified through changes in policy and practice.

What would a ‘biological turn’ in the medical humanities look like? Let's consider why older people cope less well with stress. This can only be understood when biological, psychological and social data are combined (Whalley, 2002[2001]: 94), and I argue that acknowledgement of the biological facts can help enable a focus on psychological and social elements that are at the heart of the medical humanities.^10^ The human stress response is controlled by the brain and delivered by cortisol; a hierarchy of cerebral hormones stimulates the suprarenal gland to release cortisol; in a negative feedback loop, cortisol reduces further release of the two cerebral hormones. This feedback loop restricts long-term negative effects of cortisol, including muscle wasting, irregularities in sugar metabolism and salt balance, and reduced efficiency of the immune system. Ageing disrupts this negative feedback. Cortisol background levels are higher, and, following activation, return to baseline takes longer. Physiological consequences of this are changes in brain function and biological rhythms. Across the lifespan, factors like diet and lifestyle support stress management. The older we get, the more social and psychological factors complicate the control of cortisol levels; interventions at individual, community, and population levels could make a difference to health experiences in older age. Consider, for example, anxieties about ageism in the job market, alongside the growing likelihood of stressful life events (children leaving home, the death of a spouse, a health diagnosis). All these matters further challenge pre-existing biological, age-related changes in cortisol balance, but they can be contained (and health in older age maintained for longer), for example, by provision of meaningful social portfolios that address withdrawal and social isolation (Cohen, 2006[2005]: 125–30). A greater emphasis in medical humanities scholarship on biological realities would support more effective strategies for achieving shifts in social and psychological factors that age us prematurely.

## Conclusion: Striving for a culture of care in a cure society

Human cultures absorb medical actualities. Specific periods are associated with particular kinds of medicine, and this medicine, through mechanisms of popularization and mythologization, influences literary writings (Rousseau, 1991: 6). I would go further and argue that particular kinds of science or medicine (for example, a disease model, a visualizing technique, or a research hypothesis), which capture the popular imagination at a given time, can also direct critical scholarship. This means that long-established, old-fashioned, or less captivating research paths may become overlooked or undervalued.

In age studies, field-defining work has challenged the ‘biological essentialism projected onto old age – that one would inevitably age into frailty, dementia, and loneliness’ (Jewusiak, 2023: 3). Challenging this decline narrative is indispensable, way beyond concerns about negative stereotype embodiment. It is crucial in a cure-focused context. Where a cure is not forthcoming, as in chronic degenerative conditions like Alzheimer's, Parkinson's, and arthritis, ageing is seen as a failure with significant implications for non-care or un-care. The absence of effective treatment, according to Robertson and Travaglia, ‘negates the capabilities of medicine as an interventional form of practice’, which means that disease management becomes the only option. But in a performance- and profit-driven society, this leads to ‘rationing care’ for the frail, vulnerable, and incurable, because the old person, with or without a degenerative condition, is on an ‘inevitable pathway’ of decline and towards death (Robertson and Travaglia, 2023: 137–8; see also Bunting, 2021[2020]). In this context, a critique of the biomedical as representing an exclusively cure-driven focus on disease is valuable (on the duality between cure and care in healthcare and implications for chronic illnesses, cf. Pachoud in this issue).

Over the years, the range of disciplines invested in humanistic studies in ageing widened, but the ‘gap’ between humanists and gerontologists remained (Goldman, de Medeiros, and Cole, 2022: 3). To bridge this gap, *Critical Humanities and Ageing* (2022), as the fourth volume in the series of textbooks and guides to humanistic age studies, was set up, as its subtitle indicates, to ‘forge interdisciplinary dialogues’ in chapters that include a response from another discipline.^11^ This is an important development, but it does not go far enough. The medical humanities, with their strong foothold in bio-psycho-social dimensions of health and disease, can do more. They need to engage in biology directly, with a capacity to modify biological pathways.

Since ageing comes with decline and degeneration, a greater cultural presence and acknowledgement of the biological realities of lifetime ageing would be productive, especially because related research has the potential to dismantle lifetime inequalities. Precisely these inequalities determine how early or late in life we see the decline of capabilities that can compensate for biological ageing, with physiological consequences and functional outcomes (Ferrucci *et al.*, 2018). Ageing along a biological continuum can be an empowering concept for improving ageing – if it is connected to work, including at policy levels, that challenges health inequalities and enables action on modifiable risk factors, particularly upstream of health inequalities.

In the context of chronic disease management, economies of resources and technologies further contribute to inequalities. As stated 20 years ago, biomedicalization has led to an expectation that the management of chronic health conditions is an individual's moral responsibility; and this has to be fulfilled by way of self-surveillance, prevention and the consumption of self-help goods and services, including fit bits and brain games (Clarke *et al.*, 2003: 162). Self-tracking technologies have turned into tools for health management, and digital self-care patterns are beginning to change the relationship between patient and physician, while also influencing self-perception. In particular, such technologies produce an ‘ambiguous image’ of the ageing body and the ageing brain: on the one hand as ‘improvable and “plastic”’, but also as ‘inevitably in decline’ (Katz and Marshall, 2018: 63). This is especially problematic for older individuals who have reached a high level of vulnerability and dependence on others (Wahl, Iwarsson, and Oswald, 2012).^12^

The experiential consequences of memory loss are devastating, and frailty and vulnerability in old age are a reminder that the reality of decline and loss cannot be ignored. Narratives about ageing as a reality – of bodily change, the potential of ill health and frailty – exist; but they have a limited audience, in medical humanities scholarship and wider culture. The biological substrates of such lived reality have the potential to be modified by intervening in social and psychological factors. In this context, narrative approaches themselves become interventions. Narrative practice is a form of care (Sako and Falcus, 2022: 4). This includes time for narrative in the clinical encounter, also as digitization increasingly directs the further marginalization of many older people, while exacerbating inequalities. Narrative approaches will continue to be essential for challenging the kind of postgenomic reductionism and stereotyping across categories of difference, including age, that come with an epigenetic focus on somatic manifestations of exposures and experiences (see e.g. Richardson, 2015). Narrative research needs to continue to remain a central element in the health sector (Greenhalgh, 2016), because it conveys and creates the meanings of the biologically ageing body and fosters appreciation that ageing is neither uniform nor predetermined. Most of all, stories create and cohere collective identities; they are essential to public reasoning (Dillon and Craig, 2021: 49–50). Narrative captures the stories about ageing we live by. These stories influence how we age, biologically, psychologically, socially; and they inform how we approach care, do research, and plan our lives. Expectations of health (or not) in older age determine how we think about our own future selves. Contextualized research will achieve healthier ageing for all, and this will generate new thinking about ageing, which, in turn, will come with downstream effects for more optimistic stories and perceptions of growing older in the future.

## Notes

Ideas presented in this manuscript were influenced by discussions in a series of workshops led by Patrick ffrench, Céline Lefève, and Neil Vickers, based on a collaboration between King’s College London and the Institut la Personne en Médecine, Université Paris Cité (King’s Global Engagement). I owe thanks for discussion to the workshop participants, Isabelle Aujoulat, Caitjan Gainty, Tania Gergel, Bernard Pachoud, Mohammed Rashed, Elise Ricadat, and Karl-Léo Schwering. Patrick ffrench, Brian Hurwitz, Céline Lefève, and Neil Vickers offered invaluable feedback on a draft manuscript.

1. I articulated these aspects as characterizing optimistic realism about ageing in a seminar held at the School of Lifecourse and Population Health Sciences, King’s College London, in June 2023, and owe thanks to Katie Sheehan and Claire Steves for discussion.

2. *The Edinburgh Companion to the Critical Medical Humanities* (2016) does not contain a chapter specifically on ageing and older age. That said, scholarship on ageing may make use of learning from the medical humanities but not necessarily refer to the field (consider e.g. reflections in Murray, 2023: 3–4).

3. An anonymous reviewer used the term ‘biological turn’, and I here integrate it into my argument.

4. Some of the points articulated in this paragraph have previously been published open access (Zimmermann, 2024: 331–2).

5. I am indebted to Jasmine Fledderjohann, Lancaster University, for discussion of Conrad’s work.

6. I owe thanks to Sridhar Venkatapuram, King’s College London, for pointing me to Jecker’s meticulous and circumspect scholarship.

7. Susan Pickard similarly considers Scott-Maxwell’s account as articulating strength in frailty (2018: 30).

8. As regards the human genome project, the expansion of knowledge about human ageing and longevity genes has remained limited (Anton, 2013); consequently, much store is put into postgenomic research as discussed further below in this article (see e.g. Barzilai and Shuldiner, 2001; Tan, Kruse, and Christensen, 2006).

9. Genome-wide association studies can identify disease susceptibility genes including the biological pathways that can become implicated in clinical care; but, among others, there are concerns that association signals mostly reflect genes with no biological relevance to disease (Tam *et al.*, 2019).

10. For details on the human stress response and ageing, see Whalley’s accessible account (2002[2001]: 82–100), on which this paragraph relies. Through an increased literacy and appreciation of biology, instead of considering only select scientific narratives, medical humanities scholars could, additionally, remove themselves from being perceived as using biology solely as an object of enquiry. The humanities have a crucial role to play in public reasoning about ageing. Differences in temporality and scale make it difficult for individuals to see the connection between the general and the particular: their own ageing (and their attitudes to getting older) and the challenges arising from population ageing. The humanities have the power to help people see how their experience (which happens in the present) relates to the stories told by scientists (which are about the future, and the impact of the past on the future; Dillon and Craig, 2021: 105).

11. I also note the recent *Gerontologist* special issue that explores ‘overlapping humanities–gerontology pathways that invite their various subfields and networks to collide and intersect with each other: the humanities inside gerontology and gerontology inside the humanities’ (Chivers, Katz, and Kriebernegg, 2023: 1573).

12. But note that especially media accounts of the crisis of care hardly mention relational aspects implicated in the ability of such technologies to sustain life (DeFalco, 2023: 5).
